# Síndrome de Takotsubo em Adolescente em Tratamento Oncológico: Um Raro Relato de Caso

**DOI:** 10.36660/abc.20250380

**Published:** 2026-03-26

**Authors:** Tami Inada, Guilherme Santos Marques da Silva, Raisa Machado Amaral, Edgard Ferreira dos Santos, Carlos Eduardo de Barros Branco

**Affiliations:** 1 A. C. Camargo Cancer Center São Paulo SP Brasil A. C. Camargo Cancer Center, São Paulo, SP – Brasil; 2 Instituto Universitario Escuela de Medicina del Hospital Italiano de Buenos Aires Buenos Aires Argentina Instituto Universitario Escuela de Medicina del Hospital Italiano de Buenos Aires, Buenos Aires – Argentina; 3 Hospital do Coração São Paulo SP Brasil Hospital do Coração, São Paulo, SP – Brasil; 4 Hospital Israelita Albert Einstein São Paulo SP Brasil Hospital Israelita Albert Einstein, São Paulo, SP – Brasil; 5 Instituto do Coração do Hospital das Clínicas da Faculdade de Medicina da Universidade de São Paulo São Paulo SP Brasil Instituto do Coração do Hospital das Clínicas da Faculdade de Medicina da Universidade de São Paulo, São Paulo, SP – Brasil

**Keywords:** Cardiomiopatia de Takotsubo, Adolescente, Oncologia

## Introdução

A Síndrome de Takotsubo (STT) ou cardiomiopatia de Takotsubo, foi descrita pela primeira vez no Japão em 1990 e se caracteriza por uma disfunção transitória e aguda do ventrículo esquerdo (VE), usualmente com hipocinesia ou acinesia dos segmentos médio-apicais do VE e hipercinesia dos segmentos basais. A morfologia resultante, de balonamento apical, remete a uma armadilha de polvo ("tako-tsubo"), Apesar de seus sintomas serem semelhantes aos da síndrome coronariana aguda, não há obstrução coronariana, e a alteração da motilidade ventricular costuma ultrapassar o território de uma artéria específica.^1,2^

Diversos fatores podem precipitar a STT, destacando-se gatilhos físicos (infecções graves, intervenções cirúrgicas) e emocionais. Em pacientes oncológicos, a incidência é maior, atribuída à toxicidade dos tratamentos, complicações infecciosas subsequentes e sobrecarga física e psicológica.^3,4^ A literatura aponta que pacientes oncológicos apresentam maior mortalidade e eventos cardiovasculares adversos maiores combinados quando desenvolvem STT, especialmente quando comparados àqueles sem histórico de câncer.^5^

Descrevemos um caso raro de STT em uma adolescente com tumor de células germinativas supraselar, desenvolvida após uma infecção grave e choque séptico. O objetivo é explorar a associação entre STT e condições oncológicas, destacando o pior prognóstico da STT na presença de neoplasia, além de discutir o papel de gatilhos não emocionais, como infecções graves, no desenvolvimento desta condição rara em pacientes pediátricos.

## Relato de Caso

Adolescente de 14 anos, sexo feminino, com tumor de células germinativas supraselar, em tratamento quimioterápico com carboplatina e etoposídeo. A paciente havia sido submetida a duas intervenções cirúrgicas para ressecção de tumor hipofisário. A primeira, realizada por via endoscópica em 02/12/2019, teve resultado de análise anatomopatológica inconclusivo. A segunda cirurgia, por via aberta, ocorreu em 07/02/2020, resultando em ressecção incompleta; o exame anatomopatológico revelou neoplasia maligna indiferenciada. No seguimento, apresentou disfunções endócrinas secundárias ao comprometimento tumoral, incluindo diabetes insípido, hipotireoidismo e insuficiência adrenal, todas controladas com terapia de reposição hormonal: levotiroxina 25 mcg/dia e desmopressina (DDAVP) *spray* nasal, dois jatos/dia.

A paciente deu entrada hospitalar com neutropenia febril, 13 dias após o segundo ciclo de quimioterapia. Iniciou cefepima, evoluindo com diarreia associada à *Clostridium difficile*, hipernatremia e desidratação, sendo introduzido metronidazol. No exame físico inicial, os sinais vitais incluíam frequência cardíaca de 91 bpm e pressão arterial de 110 × 79 mm Hg. O peso era de 66 kg e a altura de 159 cm. A paciente encontrava-se em bom estado geral, descorada (+/4+), hemodinamicamente estável, eupneica, ativa e contactante, porém sem abertura ocular espontânea. Apresentava ritmo cardíaco regular em dois tempos, bulhas normofonéticas, sem sopros. A ausculta pulmonar revelava murmúrio vesicular presente bilateralmente, sem ruídos adventícios. O abdome era plano, flácido, indolor à palpação, sem visceromegalias. As extremidades apresentavam perfusão periférica preservada, sem edema ou cianose.

No terceiro dia, apresentou febre alta, rebaixamento do nível de consciência e choque séptico. Foi encaminhada à unidade de terapia intensiva pediátrica, intubada, com acesso central e infusão de adrenalina, corticoterapia (prednisona 5 mg) e antibioticoterapia ampliada (Vancomicina e Meropenem). O exame neurológico à admissão evidenciava paciente vígil, responsiva ao comando verbal, porém sem abertura ocular espontânea, sugerindo rebaixamento do nível de consciência. Não foram observados sinais de déficit motor focal em membros à propedêutica inicial. A tomografia de crânio, não evidenciou alterações estruturais; hemoculturas foram negativas.

**Figura 1:**
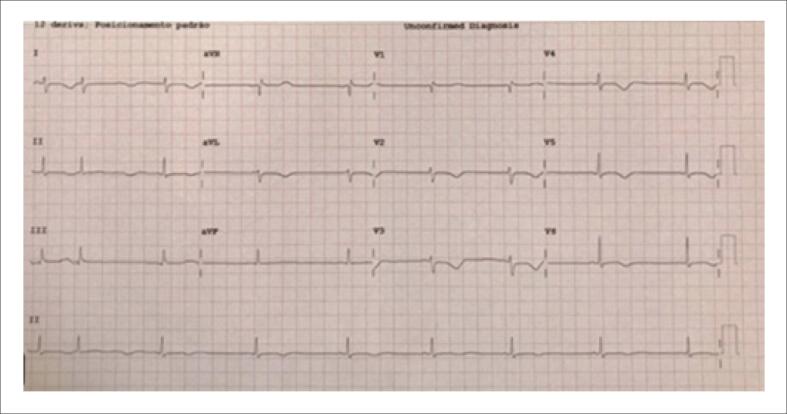
Inversão da onda T e prolongamento do intervalo QT.

**Figura 2:**
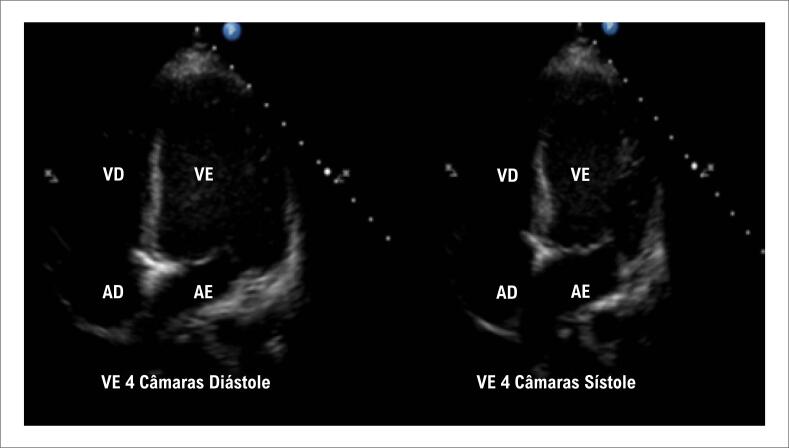
Ecocardiograma mostrando acinesia médio-apical do ventrículo esquerdo. VD: ventrículo direito; VE: ventrículo esquerod; AD: átrio direito; AE: átrio esquerdo.

Durante a internação, registrou-se elevação dos níveis séricos de troponina, associada a alterações eletrocardiográficas caracterizadas por inversão da onda T e prolongamento do intervalo QT (Figura 1). O ecocardiograma revelou acinesia dos segmentos médio-apicais do VE, com fração de ejeção do VE (FEVE) de 29% e função ventricular direita preservada (Figura 2). A paciente recebeu milrinona (0,5 mcg/kg/min) para suporte inotrópico. No dia seguinte, apresentou parada cardiorrespiratória por fibrilação ventricular, revertida após manobras de ressuscitação cardiopulmonar. O suporte inotrópico foi ajustado, substituindo-se milrinona por dobutamina (3,5 mcg/kg/min).

A suspeita de STT foi confirmada pela recuperação da função ventricular. A miocardite foi descartada e não houve evidência de obstrução coronariana. Iniciou-se tratamento com carvedilol e enalapril.

A paciente evoluiu com melhora clínica; foi extubada ao sétimo dia, suspendeu inotrópicos ao oitavo dia, e apresentou normalização da FEVE (62%) ao nono dia. No 10° dia de internação, a paciente encontrava-se afebril (36,2–36,6 °C), com frequência cardíaca entre 48 e 86 bpm, frequência respiratória de 15 a 21 irpm, pressão arterial média entre 82 e 150 mmHg e saturação periférica de oxigênio de 95–98% em ar ambiente. A diurese no período diurese foi de 686 mL, com evacuação semilíquida e balanço hídrico negativo de –262 mL.

Ao exame físico, apresentava-se em regular estado geral, discretamente descorada, normo-hidratada, com escala de Glasgow 15. Observou-se estrabismo divergente e anisocoria à direita. O murmúrio vesicular estava presente bilateralmente, sem ruídos adventícios. A ausculta cardíaca revelava bulhas normofonéticas, sem sopros, frequência cardíaca de 65 bpm e pressão arterial média de 86 mmHg. O abdome encontrava-se flácido, com ruídos hidroaéreos presentes e sem visceromegalias palpáveis. Pulsos periféricos estavam cheios, tempo de enchimento capilar de 2 segundos e extremidades aquecidas, bem perfundidas.

**Figura 3:**
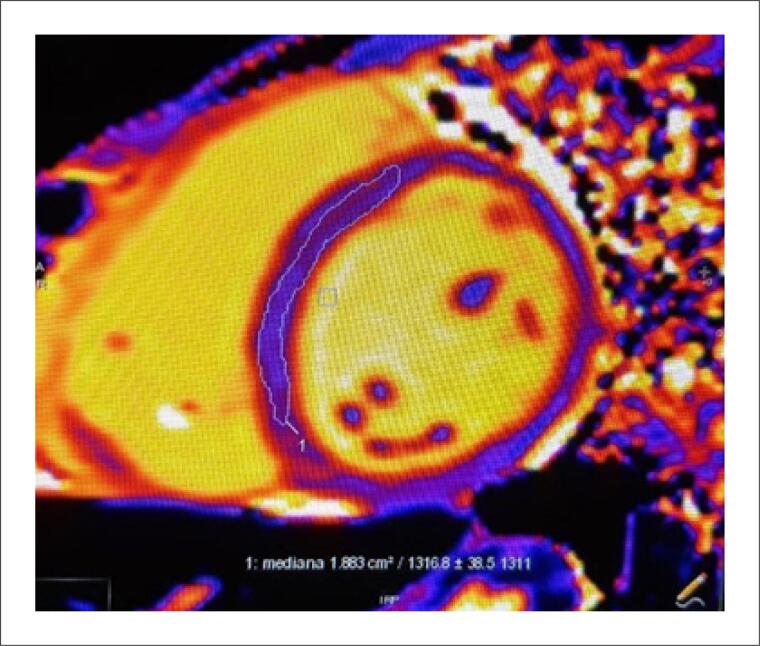
Ressonância magnética cardíaca mostrando função sistólica biventricular preservada e discreto realce não isquêmico nos segmentos médio-basais da parede inferior do ventrículo esquerdo, evidenciado no T1 mapping. Não se observa padrão típico de realce tardio de gadolínio com anulação do miocárdio.

A ressonância magnética no 10° dia evidenciou função biventricular normal, com realce tardio discreto não isquêmico (Figura 3). No 15° dia de internação, a paciente encontrava-se em bom estado geral, hipocorada (+/4+), anictérica, acianótica, afebril e emagrecida (+/4+), com melhora progressiva da abertura palpebral direita. À ausculta respiratória, observa-se murmúrio vesicular globalmente diminuído, sem ruídos adventícios. A ausculta cardíaca evidenciava bulhas normofonéticas em dois tempos, sem sopros. O Abdome apresentava-se plano, flácido, indolor, com ruídos hidroaéreos presentes. Avaliação neurológica sem sinais meníngeos, com adequada perfusão periférica.

No período, não foram registrados novos episódios de diarreia, o sódio sérico manteve-se estável e tanto a pressão arterial quanto a frequência cardíaca permaneceram normais. A paciente recebeu alta para a enfermaria mantendo enalapril, carvedilol e prednisona 5 mg/dia, além da terapia de reposição hormonal com levotiroxina 25 mcg/dia e DDAVP spray nasal, dois jatos/dia.

## Discussão

A STT é uma cardiomiopatia transitória caracterizada por disfunção sistólica segmentar do VE, usualmente com acinesia ou hipocinesia dos segmentos médio-apicais e hipercinesia compensatória dos segmentos basais, em ausência de obstrução coronariana significativa.^1,2^ Embora classicamente associada a estressores emocionais em mulheres pós-menopáusicas, a STT tem sido cada vez mais reconhecida em contextos clínicos diversos, incluindo pacientes jovens, oncológicos e críticos, ampliando seu espectro epidemiológico.

Neste relato, descrevemos uma paciente pediátrica com neoplasia de células germinativas da região supra-selar, submetida a tratamento quimioterápico intensivo e portadora de disfunções endócrinas, que evoluiu com STT em vigência de choque séptico. O quadro clínico remete à forma clássica da síndrome, mas destaca uma população raramente descrita na literatura: crianças imunocomprometidas com doenças graves de base.

Embora formas variantes – como as apresentações midventricular, basal, invertida ou focal – também estejam descritas, a apresentação biventricular, observada em menos de 0,5% dos casos, cursa com pior prognóstico hemodinâmico.^3,6^ A associação entre infecção por Clostridium, instabilidade circulatória e disfunção miocárdica reforça o papel dos gatilhos físicos, em especial a sepse, como indutores da cascata fisiopatológica da STT em pacientes vulneráveis.

A classificação dos gatilhos em emocionais e não emocionais, proposta na literatura, permite compreender melhor os mecanismos subjacentes à ativação adrenérgica. Em crianças, eventos infecciosos e neurológicos parecem predominar como fatores precipitantes, conforme descrito por Finsterer et al.^7^ O caso apresentado é condizente com essa observação, em que a sepse e o uso de inotrópicos atuaram como possíveis catalisadores da síndrome.

Relatos prévios, como o descrito por Firdouse et al.,^8^ reforçam que, embora infrequente, a STT pode ocorrer em pediatria e, quando diagnosticada precocemente, tende à recuperação ventricular completa.^9^ O International Takotsubo Registry, que envolveu mais de 1700 pacientes, demonstrou predominância feminina (89,8%) e idade média de 66,8 anos, com gatilhos físicos superando os emocionais (36% versus 27,7%). Curiosamente, em quase um terço dos casos, nenhum gatilho claro foi identificado.^3^

No cenário oncológico, a STT tem sido descrita com maior frequência em pacientes com neoplasias sólidas avançadas, notadamente tumores de mama, trato gastrointestinal e pulmão.^10^ Essa associação está presente no presente caso, evidenciando a inter-relação entre estresse físico, inflamação sistêmica e exposição a agentes cardiotóxicos. A literatura estima que até 6,6% dos pacientes com STT possam ter diagnóstico concomitante de câncer.^10^

Diversos agentes quimioterápicos foram implicados como potenciais indutores da STT, entre eles o 5-fluorouracil, conforme descrito por Ozturk et al., e o etoposídeo, citado por Escoto et al. em pacientes pediátricos.^11,12^ A toxicidade miocárdica pode decorrer tanto de efeitos diretos das drogas quanto de resposta exacerbada do eixo neuro-hormonal ao estresse clínico.

Do ponto de vista fisiopatológico, a síndrome é atribuída à ativação excessiva do sistema nervoso simpático, com liberação de catecolaminas em níveis tóxicos ao miocárdio, induzindo espasmo coronariano, disfunção microvascular e atordoamento miocárdico.^8,13^ Apesar da presença de discreto realce tardio com padrão não isquêmico na ressonância magnética, não foram observados sinais de edema miocárdico nas sequências ponderadas em T2. Ademais, a rápida recuperação da função ventricular esquerda, documentada tanto pelo ecocardiograma quanto pela ressonância magnética, torna o diagnóstico de miocardite pouco provável, sustentando o diagnóstico de STT.^9^ Além dos fatores cardiovasculares diretos, evidências recentes apontam para o envolvimento do sistema nervoso central. Estudos com neuroimagem funcional sugerem alteração na conectividade entre regiões cerebrais responsáveis pela modulação do estresse e pela resposta autonômica, conferindo plausibilidade à hipótese da "ponte cérebro-coração".^14,15^

**Figura 4:**
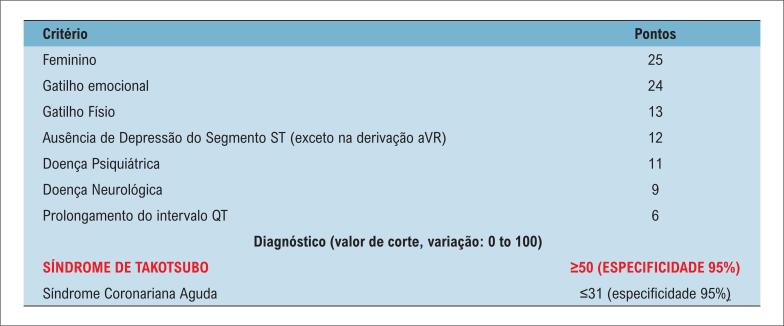
Critérios Diagnósticos InterTAK para Síndrome de Takotsubo. Fonte: Adaptado de Assad et al.^16^

No caso relatado, o desencadeante mais evidente foi a sepse, seguida por parada cardíaca em fibrilação ventricular. A administração de inotrópicos como adrenalina e milrinona pode ter contribuído para a amplificação do estímulo simpático, funcionando como coadjuvante no desenvolvimento da disfunção miocárdica. Os registros RETAKO e REMUTA revelam que pacientes com STT associada ao câncer apresentam maior incidência de complicações cardiovasculares, maior mortalidade hospitalar e maior frequência de eventos adversos combinados (Figura 4).^5,16^

## Conclusão

A STT, embora classicamente descrita em mulheres idosas com estressores emocionais, deve ser considerada também em pacientes oncológicos pediátricos expostos a gatilhos físicos significativos, como infecções graves e sepse. Este caso ilustra a importância da suspeição clínica em contextos atípicos e reforça a necessidade de uma abordagem diagnóstica acurada, especialmente em pacientes imunocomprometidos e com histórico de terapias potencialmente cardiotóxicas.

A escassez de dados robustos em populações pediátricas, sobretudo em crianças com neoplasias, evidencia uma lacuna importante na literatura. A implementação de registros internacionais específicos para essa faixa etária pode contribuir substancialmente para a compreensão do espectro clínico, dos mecanismos fisiopatológicos envolvidos e da resposta terapêutica nesses casos.

Por fim, o reconhecimento precoce da STT nesse perfil de paciente é fundamental não apenas para o manejo cardiológico adequado, mas também para garantir a continuidade do tratamento oncológico, minimizando riscos e otimizando desfechos clínicos.

## Data Availability

Os conteúdos subjacentes ao texto da pesquisa estão contidos no manuscrito.
