# Supplementary material for: Síndrome de Takotsubo em Adolescente em Tratamento Oncológico: Um Raro Relato de Caso
Source: Arq Bras Cardiol. 2026 Mar 26;123(2):e20250380. [Article in Portuguese] doi: 10.36660/abc.20250380 (PMC13128174; doi:10.36660/abc.20250380)
Supplement: Translation

# Translation: Takotsubo Syndrome in an Adolescent Undergoing Oncological Treatment: A Rare Case Report

**DOI:** 10.36660/abc.20250380i

**Authors:** Tami Inada, Guilherme Santos Marques da Silva, Raisa Machado Amaral, Edgard Ferreira dos Santos, Carlos Eduardo de Barros Branco

**Affiliations:** 1 São Paulo SP Brazil A. C. Camargo Cancer Center, São Paulo, SP – Brazil; 2 Buenos Aires Argentina Instituto Universitario Escuela de Medicina del Hospital Italiano de Buenos Aires, Buenos Aires – Argentina; 3 São Paulo SP Brazil Hospital do Coração, São Paulo, SP – Brazil; 4 São Paulo SP Brazil Hospital Israelita Albert Einstein, São Paulo, SP – Brazil; 5 São Paulo SP Brazil Instituto do Coração do Hospital das Clínicas da Faculdade de Medicina da Universidade de São Paulo, São Paulo, SP – Brazil

**Keywords:** Takotsubo Cardiomyopathy, Adolescent, Medical Oncology

## Introduction

Takotsubo Syndrome (TTS), or Takotsubo cardiomyopathy, was first described in Japan in 1990 and is characterized by transient and acute dysfunction of the left ventricle, usually with hypokinesia or akinesia of the mid-apical LV segments and hyperkinesia of the basal segments. The resulting morphology, with apical ballooning, resembles an octopus trap ("tako-tsubo"). Despite its symptoms being similar to those of acute coronary syndrome, there is no coronary obstruction, and the ventricular wall motion abnormality usually extends beyond the territory of a single artery.^1,2^

Several factors can precipitate TTS, particularly physical triggers (such as severe infections and surgical interventions) and emotional stress. In oncology patients, the incidence is higher, attributed to treatment-related toxicity, subsequent infectious complications, and physical and psychological overload.^3,4^ The literature indicates that oncology patients present higher mortality and more combined major adverse cardiac events when they develop TTS, especially compared to those without a history of cancer.^5^

We describe a rare case of TTS in an adolescent with a suprasellar germ cell tumor, which developed after a severe infection and septic shock. The objective is to explore the association between TTS and oncological conditions, highlighting the worse prognosis of TTS in the presence of neoplasia, as well as to discuss the role of non-emotional triggers, such as severe infections, in the development of this rare condition in pediatric patients.

## Case Report

A 14-year-old female adolescent with a suprasellar germ cell tumor, undergoing chemotherapy with carboplatin and etoposide. The patient had previously undergone two surgical procedures for resection of a pituitary tumor. The first, performed via endoscopic approach on 12/02/2019, yielded inconclusive histopathological results. The second surgery, performed via open approach on 02/07/2020, resulted in incomplete resection; the histopathological examination revealed an undifferentiated malignant neoplasm. During follow-up, she developed endocrine dysfunctions secondary to tumor involvement, including diabetes insipidus, hypothyroidism, and adrenal insufficiency, all controlled with hormone replacement therapy: levothyroxine 25 mcg/day and desmopressin (DDAVP) nasal spray, two sprays/day.

The patient was admitted to the hospital with febrile neutropenia, 13 days after the second cycle of chemotherapy. Cefepime was initiated, and she subsequently developed diarrhea associated with Clostridium difficile, hypernatremia, and dehydration, leading to the introduction of metronidazole. On initial physical examination, vital signs included a heart rate of 91 bpm and blood pressure of 110 × 79 mmHg. Her weight was 66 Kg and height 159 cm. The patient was in good general condition, pale (+/4+), hemodynamically stable, eupneic, active, and responsive, but without spontaneous eye opening. Cardiac examination revealed a regular rhythm in two beats, normal heart sounds, and no murmurs. Pulmonary auscultation showed bilateral vesicular breath sounds, without adventitious noises. The abdomen was flat, flaccid, non-tender on palpation, and without visceromegaly. The extremities had preserved peripheral perfusion, with no edema or cyanosis.

On the third day, she developed high fever, decreased level of consciousness, and septic shock. She was transferred to the pediatric intensive care unit, intubated, with central venous access, adrenaline infusion, corticosteroid therapy (prednisone 5 mg), and broad-spectrum antibiotic therapy (vancomycin and meropenem). Neurological examination at admission showed the patient was alert and responsive to verbal commands, but without spontaneous eye opening, suggesting impaired consciousness. No signs of focal motor deficit in the limbs were observed on initial assessment. Cranial CT scan revealed no structural abnormalities; blood cultures were negative.

During hospitalization, elevated serum troponin levels were recorded, associated with electrocardiographic changes characterized by T-wave inversion and QT interval prolongation (Figure 1). Echocardiography revealed akinesia of the mid-apical left ventricular segments, with a left ventricular ejection fraction (LVEF) of 29% and preserved right ventricular function (Figure 2).

**Figure 1:** T-wave inversion and QT interval prolongation.

**Figure 2:** Echocardiogram showing mid-apical akinesia of the left ventricle. RV: right ventricle; LV: left ventricle; RA: right atrium; LA: left atrium.

The patient received milrinone (0.5 mcg/kg/min) for inotropic support. The following day, she experienced cardiopulmonary arrest due to ventricular fibrillation, which was reversed after cardiopulmonary resuscitation (CPR). Inotropic support was adjusted, replacing milrinone with dobutamine (3.5 mcg/kg/min).

The suspicion of TTS was confirmed by recovery of ventricular function. Myocarditis was ruled out, and there was no evidence of coronary obstruction. Treatment with carvedilol and enalapril was initiated.

During hospitalization, the patient showed clinical improvement; she was extubated on the seventh day, discontinued inotropic support on the eighth day, and demonstrated normalization of LVEF (62%) on the ninth day. On the 10th day of hospitalization, the patient was afebrile (36.2–36.6 °C), with heart rate ranging from 48 to 86 bpm, respiratory rate between 15 and 21 breaths per minute, mean arterial pressure between 82 and 150 mmHg, and peripheral oxygen saturation of 95–98% on room air. Urine output during the period was 686 mL, with semiliquid stool and a negative fluid balance of −262 mL.

On physical examination, she was in fair general condition, mildly pale, normohydrated, with a Glasgow Coma Scale score of 15. Divergent strabismus and right anisocoria were observed. Pulmonary auscultation revealed bilateral vesicular breath sounds, without adventitious noises. Cardiac auscultation showed normal heart sounds, no murmurs, heart rate of 65 bpm, and mean arterial pressure of 86 mmHg. The abdomen was flaccid, with present bowel sounds and no palpable visceromegaly. Peripheral pulses were full, capillary refill time was 2 seconds, and extremities were warm and well perfused.

On the 10th day, magnetic resonance imaging showed normal biventricular function, with mild non-ischemic late enhancement (Figure 3). On the 15th day of hospitalization, the patient was in good general condition, pale (+/4+), anicteric, acyanotic, afebrile, and underweight (+/4+), with progressive improvement of right eyelid opening. Respiratory auscultation revealed globally diminished vesicular breath sounds, without adventitious noises. Cardiac auscultation demonstrated normal heart sounds in two beats, without murmurs. The abdomen was flat, flaccid, non-tender, with present bowel sounds. Neurological evaluation showed no meningeal signs, with adequate peripheral perfusion.

**Figure 3:** Cardiac magnetic resonance imaging showing preserved biventricular systolic function and mild non-ischemic enhancement in the mid-basal segments of the inferior wall of the left ventricle, demonstrated on T1 mapping. No typical pattern of late gadolinium enhancement with myocardial nulling was observed.

During this period, no new episodes of diarrhea were recorded, serum sodium levels remained stable, and both blood pressure and heart rate stayed within normal ranges. The patient was discharged to the ward while continuing enalapril, carvedilol, and prednisone 5 mg/day, in addition to hormone replacement therapy with levothyroxine 25 mcg/day and DDAVP nasal spray, two sprays/day.

## Discussion

TTS is a transient cardiomyopathy characterized by segmental systolic dysfunction of the left ventricle, usually with akinesia or hypokinesia of the mid-apical segments and compensatory hyperkinesia of the basal segments, in the absence of significant coronary obstruction.^1,2^ Although classically associated with emotional stressors in postmenopausal women, TTS has increasingly been recognized in diverse clinical contexts, including young, oncological, and critically ill patients, thereby expanding its epidemiological spectrum.

In this case report, we describe a pediatric patient with a suprasellar germ cell neoplasm, undergoing intensive chemotherapy and presenting with endocrine dysfunctions, who developed TTS in the setting of septic shock. The clinical presentation resembles the classical form of the syndrome but highlights a population rarely described in the literature: immunocompromised children with severe underlying diseases.

Although variant forms – such as midventricular, basal, inverted, or focal presentations – have also been described, the biventricular presentation, observed in less than 0.5% of cases, is associated with worse hemodynamic prognosis.^3,6^ The association between Clostridium infection, circulatory instability, and myocardial dysfunction reinforces the role of physical triggers, particularly sepsis, as inducers of the pathophysiological cascade of TTS in vulnerable patients.

The classification of triggers into emotional and non-emotional, as proposed in the literature, allows for a better understanding of the mechanisms underlying adrenergic activation. In children, infectious and neurological events appear to predominate as precipitating factors, as described by Finsterer et al.^7^ The case presented is consistent with this observation, in which sepsis and the use of inotropes acted as possible catalysts of the syndrome.

Previous reports, such as the one described by Firdouse et al.,^8^ reinforce that although infrequent, TTS can occur in pediatric patients and, when diagnosed early, tends to result in complete ventricular recovery.^9^ The International Takotsubo Registry, which included more than 1,700 patients, demonstrated a predominance of females (89.8%) and a mean age of 66.8 years, with physical triggers surpassing emotional ones (36% versus 27.7%). Interestingly, in nearly one-third of cases, no clear trigger was identified.^3^

In the oncological setting, TTS has been described more frequently in patients with advanced solid tumors, notably breast, gastrointestinal, and lung cancers.^10^ This association is evident in the present case, highlighting the interplay between physical stress, systemic inflammation, and exposure to cardiotoxic agents. The literature estimates that up to 6.6% of patients with TTS may have a concomitant diagnosis of cancer.^10^

Several chemotherapeutic agents have been implicated as potential inducers of TTS, among them 5-fluorouracil, as described by Ozturk et al., and etoposide, reported by Escoto et al. in pediatric patients.^11,12^ Myocardial toxicity may result both from the direct effects of these drugs and from an exacerbated neurohormonal axis response to clinical stress.

From a pathophysiological perspective, the syndrome is attributed to excessive activation of the sympathetic nervous system, with catecholamine release at levels toxic to the myocardium, inducing coronary spasm, microvascular dysfunction, and myocardial stunning.^8,13^ Despite the presence of mild non-ischemic late enhancement on cardiac magnetic resonance imaging, no signs of myocardial edema were observed in T2-weighted sequences. Moreover, the rapid recovery of left ventricular function, documented both by echocardiography and magnetic resonance imaging, makes the diagnosis of myocarditis unlikely, supporting the diagnosis of TTS.^9^ In addition to direct cardiovascular factors, recent evidence points to the involvement of the central nervous system. Functional neuroimaging studies suggest altered connectivity between brain regions responsible for stress modulation and autonomic response, lending plausibility to the "brain-heart bridge" hypothesis.^14,15^

In the reported case, the most evident trigger was sepsis, followed by cardiac arrest due to ventricular fibrillation. The administration of inotropes such as adrenaline and milrinone may have contributed to the amplification of sympathetic stimulation, acting as a cofactor in the development of myocardial dysfunction. The RETAKO and REMUTA registries reveal that patients with TTS associated with cancer present a higher incidence of cardiovascular complications, increased in-hospital mortality, and a greater frequency of combined adverse events (Figure 4).^5,16^

**Figure 4:** InterTAK Diagnostic Criteria for Takotsubo Syndrome. Adapted by Assad et al.

## Conclusion

TTS, although classically described in elderly women with emotional stressors, should also be considered in pediatric oncology patients exposed to significant physical triggers such as severe infections and sepsis. This case illustrates the importance of clinical suspicion in atypical contexts and reinforces the need for an accurate diagnostic approach, especially in immunocompromised patients with a history of potentially cardiotoxic therapies.

The scarcity of robust data in pediatric populations, particularly in children with neoplasms, highlights an important gap in the literature. The implementation of international registries specific to this age group could substantially contribute to the understanding of the clinical spectrum, the underlying pathophysiological mechanisms, and the therapeutic response in such cases.

Finally, early recognition of TTS in this patient profile is crucial not only for appropriate cardiological management but also to ensure the continuity of oncological treatment, minimizing risks and optimizing clinical outcomes.

## Data Availability

The underlying content of the research text is contained within the manuscript.
